# Supplementary material for: Introduction to Skin Cancer: A Video Module
Source: MedEdPORTAL. 2016 Aug 5;12:10431. doi: 10.15766/mep_2374-8265.10431 (PMC6464471; doi:10.15766/mep_2374-8265.10431)
Supplement: Supplementary file 1 — A. Skin Cancer Learner Guide.pdf B. Video 1- Intro to Skin Cancer.mp4 C. Video 2- Keratinocyte Skin Cancer.mp4 D. Video 3- Overview of Pigmented Lesions.mp4 E. Video 4- ABCDE and Melanoma.mp4 F. Skin Cancer Annotated Slides.pdf G. Skin Cancer Self-Assessment.pdf [file mep-12-10431-s001.zip › F. Skin Cancer Annotated Slides.pdf]

Authors: Jasmine Rana[1] BA & Arash Mostaghimi[2] MD, MPA, MPH
[1]Harvard Medical School: jasmine_rana@hms.harvard.edu
[2]Brigham and Women's Hospital, Harvard Medical School: amostaghimi@bwh.harvard.edu

Introduction to Skin Cancer: A Video Module

Annotated Slides

Introduction to skin cancer

Jasmine Rana[1], BA
Arash Mostaghimi[2], MD, MPA, MPH
[1]Harvard Medical School: jasmine_rana@hms.harvard.edu
[2]Brigham and Women's Hospital, Harvard Medical School: amostaghimi@bwh.harvard.edu

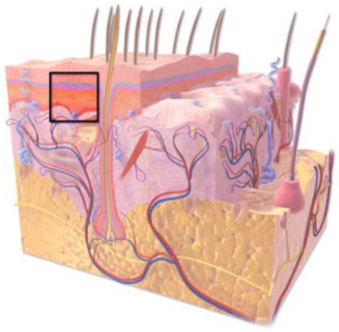

1

Let's start with the basics…

Here we are looking at a cartoon histological slice of the skin. Recall the three main layers of the skin (i.e. from the top to bottom: epidermis, dermis, and subcutaneous fat). The most common types of skin cancers we are going to focus on originate from specific cell types in the epidermis…so let's zoom in…

Authors: Jasmine Rana[1] BA & Arash Mostaghimi[2] MD, MPA, MPH
[1]Harvard Medical School: jasmine_rana@hms.harvard.edu
[2]Brigham and Women's Hospital, Harvard Medical School: amostaghimi@bwh.harvard.edu

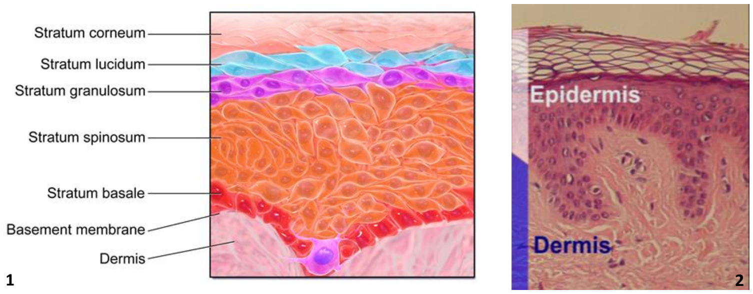

Here we are looking purely at the epidermis (cartoon on left, Hematoxylin & Eosin/"H&E stain" on right) with the basal stem cell layer at the bottom in red on the cartoon (dermis is below it) and the stratum corneum at the top in peach color on the cartoon (essentially dead keratinocytes filled with keratin).

So, how do we classify the most common types of skin cancer*? The first division is **whether it originates from keratinocytes or melanocytes (the pigment producing cells).** Recall that keratinocytes form ~ 90% of the epidermis and can be in any of the epidermal layers (basal, spinosum, granulosum, lucidum, and corneum). Melanocytes are normally found at the dermal-epidermal junction in a linear arrangement in the basal cell layer.

Here's how classification of skin cancer works:

- **We call cancers originating from melanocytes → melanoma** – simple enough!
- **Cancers originating from keratinocytes → keratinocyte carcinoma**; we further sub-classify these into:
    - Basal cell carcinoma (BCC) if it originates from the basal layer of keratinocytes
    - Squamous cell carcinoma (SCC) if it originates from the suprabasal keratinocyte cell layers

BCC and SCC can be distinguished in clinical appearance owing to different histological characteristics. In general though, these are often lumped together as "keratinocyte carcinomas" because treatment is similar. "NMSC" (non-melanoma skin cancer) is another term used for "keratinocyte carcinoma" to distinguish it from melanoma skin cancer, but the "NMSC" term is going out of vogue in order to recognize other rarer NMSC like Merkel Cell Carcinoma (neuroendocrine origin).

Epidemiologically, keratinocyte cancers account for ~97% of all skin cancers (the "most common" type of skin cancer) [1]. In general, keratinocyte carcinomas have a low metastasis rate and high cure rate if caught early (SCC has a higher rate of metastasis than BCC). While its prevalence relative to keratinocyte carcinoma is rather small (~ <2% of all skin cancer), melanoma accounts for ~80% of skin cancer deaths [2] owing to early/aggressive metastatic disease. UV exposure + fair skin complexion are major risk factors for ALL skin cancer. Smoking and HPV (human papilloma virus) are other established risk factors for SCC in particular, especially in non-sun exposed areas.

References:

[1] DeVita VT, Lawrence TS, Rosenberg SA. *DeVita, Hellman, and Rosenberg's Cancer: Principles & Practice of Oncology*. Lippincott Williams & Wilkins; 2008.

[2] What Is Melanoma? Skin Cancer Facts | Cleveland Clinic Website. https://my.clevelandclinic.org/health/diseases_conditions/hic_Skin_Cancer/hic_the_facts_about_melanoma. Accessed March 7, 2016.

**Keep in mind that there are other, rarer types of skin cancer (e.g. Merkel Cell Carcinoma) that are not covered in this module.*

Authors: Jasmine Rana[1] BA & Arash Mostaghimi[2] MD, MPA, MPH
[1]Harvard Medical School: jasmine_rana@hms.harvard.edu
[2]Brigham and Women's Hospital, Harvard Medical School: amostaghimi@bwh.harvard.edu

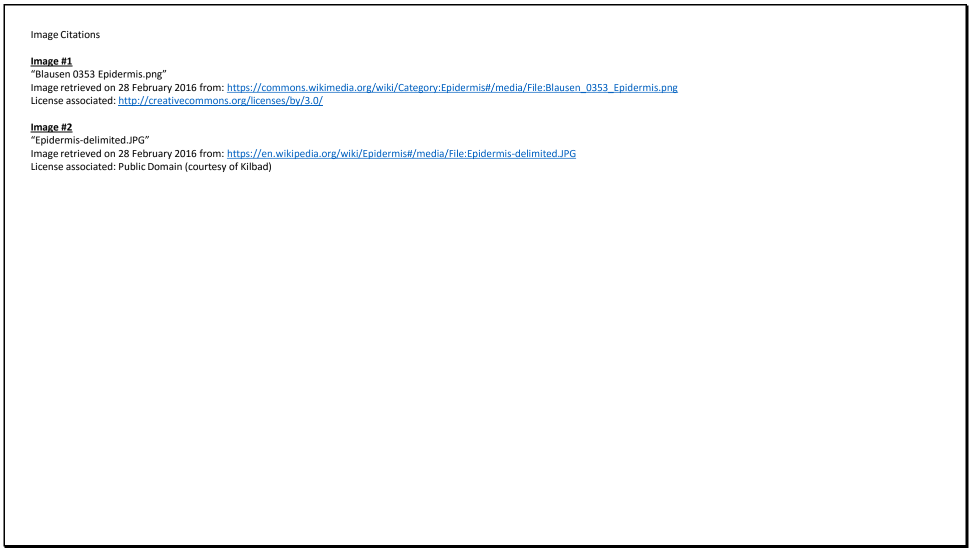

Image Citations

Image #1
"Blausen 0353 Epidermis.png"
Image retrieved on 28 February 2016 from:
https://commons.wikimedia.org/wiki/Category:Epidermis#/media/File:Blausen_0353_Epidermis.png
License associated: http://creativecommons.org/licenses/by/3.0/

Image #2
"Epidermis-delimited.JPG"
Image retrieved on 28 February 2016 from: https://en.wikipedia.org/wiki/Epidermis#/media/File:Epidermis-delimited.JPG
License associated: Public Domain (courtesy of Kilbad)

Keratinocyte skin cancer: BCC & SCC

Jasmine Rana[1], BA
Arash Mostaghimi[2], MD, MPA, MPH
[1]Harvard Medical School: jasmine_rana@hms.harvard.edu
[2]Brigham and Women's Hospital, Harvard Medical School: amostaghimi@bwh.harvard.edu

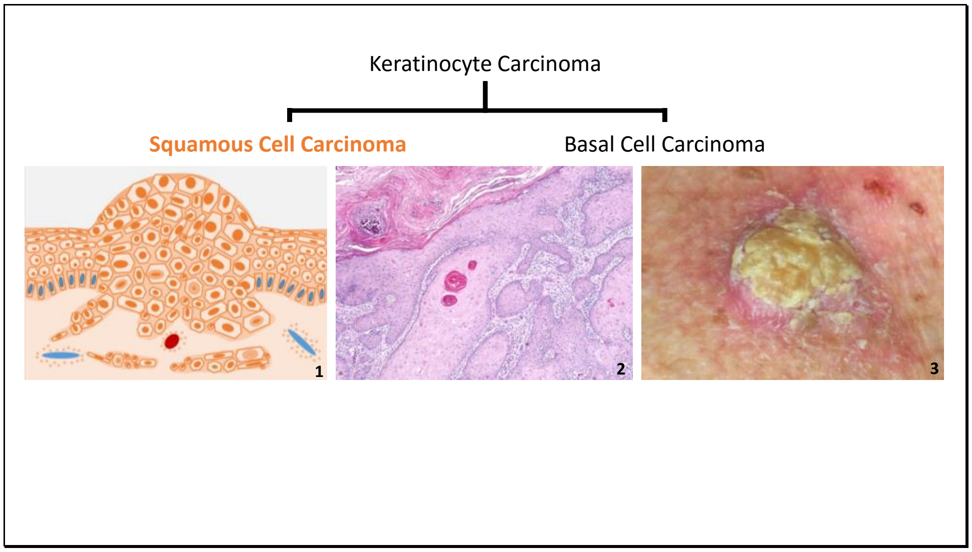

Histological-Clinical Correlation for SCC

Let's start with a cartoon histological view (left). Notice 4 key features:

- The atypical appearing keratinocytes (there is no longer orderly progression from basal cell layer to stratum corneum) = **pleomorphic** cells and nuclei (pleomorphic refers to variability in size and shape)
- Atypical cells are undergoing hyperplasia – there are way too many of them. **Acanthosis** is a dermatologic term that is often used to describe epidermal thickening like this. And the specific thickening in the most keratinized layers (corneum and granulosum) = **hyperkeratosis**.
- The key feature of invasive carcinoma is **invasion into the dermis past the basement membrane**. If there was cellular atypia without invasion into the dermis, we would call it SCC in situ (an intraepithelial neoplasm) or, if the atypia did not span the width of the epidermis, it may appear clinically as an "actinic keratosis" = a pre-malignant lesion of SCC.
- **Inflammation in dermis** (represented by speckled dots around blood vessels in cartoon image) in response to invasive cancer cells.

On H&E stain (middle panel), we again see acanthosis and hyperkeratosis of the epidermis and invasion into the dermis. Note hypereosinophilic clusters = **keratin pearls**. Remember that keratin is protein and eosin (red acidic dye in the H&E stain) binds to basic substances like proteins. Keratin pearls tend to be present in well-differentiated SCC (well-differentiated refers to the fact that cancer still closely resembles it cell of origin, in this case, the keratinocyte). As the SCC become more and more atypical and more poorly differentiated, erosion and ulceration can occur (meaning the epidermis sloughs off; **e**rosion exposes lower levels of **e**pidermis, ulceration is deeper and exposes underlying dermis). Also note that the surrounding dermal stroma is inflammatory – the hazy blue dots in dermis are aggregates of lymphocytes called into action by the inflammatory cytokines produced in the vicinity of the tumor.

Armed with this view of SCC, let's take a look at the clinical appearance of a typical SCC (right). Shown is a scaly erythematous papule/plaque on an extremity (note surrounding solar elastosis and atrophy of the skin, likely caused by chronic UV damage). What we saw as hyperkeratosis on histology appears as scale (build-up of keratin) clinically! Erythema is a reflection of vasodilation secondary to inflammation in the underlying dermis.

Authors: Jasmine Rana[1] BA & Arash Mostaghimi[2] MD, MPA, MPH
[1]Harvard Medical School: jasmine_rana@hms.harvard.edu
[2]Brigham and Women's Hospital, Harvard Medical School: amostaghimi@bwh.harvard.edu

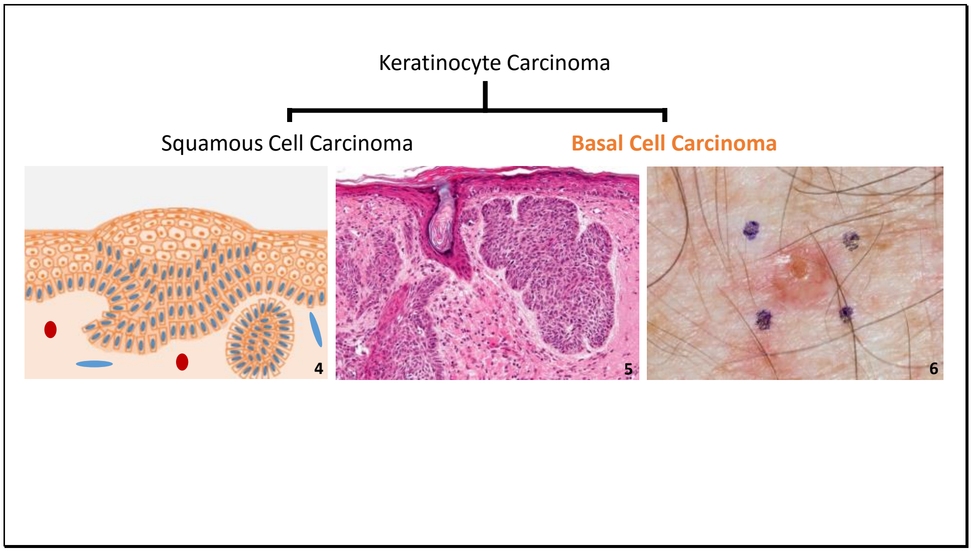

Histological-Clinical Correlation for BCC

Again, start with a cartoon histological slice of a BCC (left). Notice proliferation of the basal cell layer with prominent nuclei. Nuclei are illustrated as being blue because nuclei tend to stain blue with hematoxylin, a blue basic dye in the H&E stain that stains acidic structure like RNA and DNA in the nucleus. Again, key feature of invasive BCC is the invasion into the dermis. Notice here how the basaloid cells form "nests" of cells.

On the H&E image (middle panel) basaloid nests appear as vibrant purple-blue clusters of cells – often referred to as "basaloid nests with peripheral palisading" (n.b. the basal cell layer normally separates epidermis from dermis; in basal cell *carcinoma,* the basal cells on periphery are lining up in a neat linear fashion -- so-called "peripheral palisading" -- with the same barrier function (albeit atypically): to keep the dermal stroma away from epidermal cells). You can also appreciate stromal artifact retraction + inflammatory infiltrate. Notice how there isn't as much epidermal change as there was for SCC.

So how does this appear grossly? On the right is a typical picture of a nodular BCC (most common BCC subtype) – notice first that it is a papule with minimal scale, which makes sense: basal cells are stem cells and don't produce much keratin. BCCs also tend to be quite vascular, accounting for telangiectasias (dilated blood vessels) on the surface of lesion (note that –ectasia means "dilation"). Other typical gross features include: pearly, rolled borders, and a nodular appearance due to underlying "basaloid nests" infiltrating into the dermis. Central erosion and/or ulceration can also occur ("rodent bite ulcer") secondary to necrosis.

Authors: Jasmine Rana[1] BA & Arash Mostaghimi[2] MD, MPA, MPH
[1]Harvard Medical School: jasmine_rana@hms.harvard.edu
[2]Brigham and Women's Hospital, Harvard Medical School: amostaghimi@bwh.harvard.edu

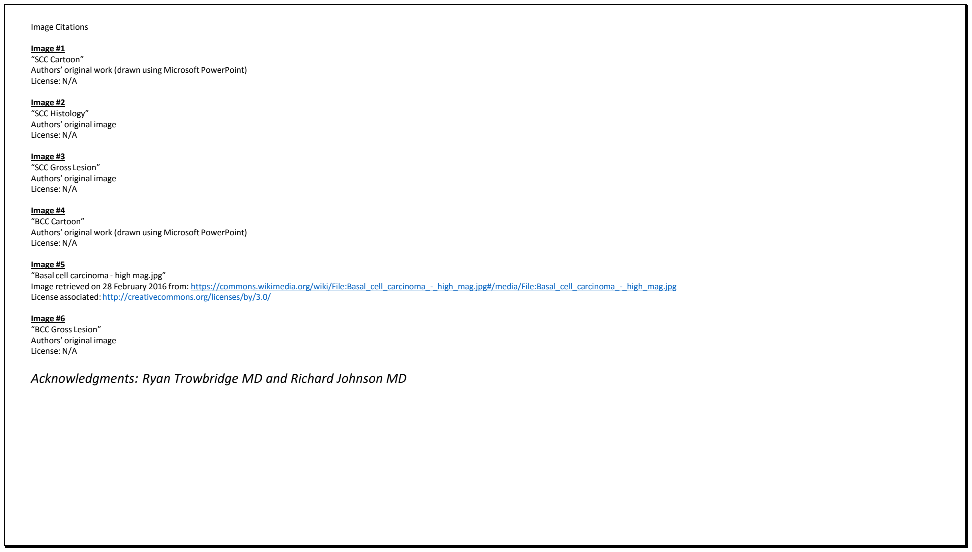

Image Citations

Image #1
"SCC Cartoon"
Authors' original work (drawn using Microsoft PowerPoint)
License: N/A

Image #2
"SCC Histology"
Authors' original image
License: N/A

Image #3
"SCC Gross Lesion"
Authors' original image
License: N/A

Image #4
"BCC Cartoon"
Authors' original work (drawn using Microsoft PowerPoint)
License: N/A

Image #5
"Basal cell carcinoma - high mag.jpg"
Image retrieved on 28 February 2016 from: https://commons.wikimedia.org/wiki/File:Basal_cell_carcinoma_-_high_mag.jpg#/media/File:Basal_cell_carcinoma_-_high_mag.jpg
License associated: http://creativecommons.org/licenses/by/3.0/

Image #6
"BCC Gross Lesion"
Authors' original image
License: N/A

Acknowledgments: Ryan Trowbridge MD and Richard Johnson MD

Overview of pigmented lesions: nevi & melanoma

Jasmine Rana[1], BA
Arash Mostaghimi[2], MD, MPA, MPH
[1]Harvard Medical School: jasmine_rana@hms.harvard.edu
[2]Brigham and Women's Hospital, Harvard Medical School: amostaghimi@bwh.harvard.edu

Authors: Jasmine Rana[1] BA & Arash Mostaghimi[2] MD, MPA, MPH
[1]Harvard Medical School: jasmine_rana@hms.harvard.edu
[2]Brigham and Women's Hospital, Harvard Medical School: amostaghimi@bwh.harvard.edu

DEFINITIONS

Benign nevi // Dysplastic nevi // Melanoma

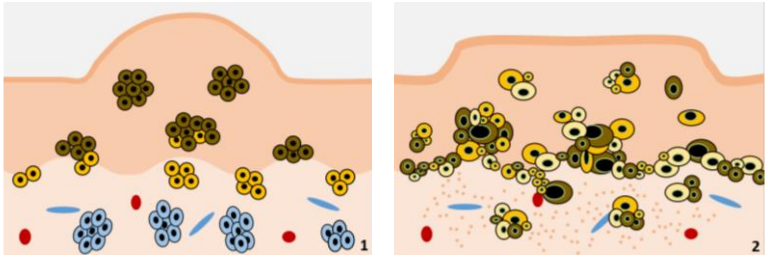

You might hear terms like "nevi", "dysplastic nevi", and "melanoma" and know that they all have something to do with pigment-producing cells, but what distinguishes them? And what exactly is a nevus/nevi referring to? Nevus cells are variants of melanocytes (distinction isn't terribly important for our purposes and we will refer to them as melanocytes) and they are the primary component of a melanocytic nevus (mole), which is why we call moles "nevi" (pleural).

Notice that pigmented lesions are written suggestively from benign to most malignant to show they are all on a spectrum with dysplastic (irregular) nevi somewhere in between, but there are NO arrows suggesting that benign lesions "turn into" more malignant lesions. While some dysplastic nevi may transform into melanoma, there is not a well-understood pathophysiological causal pathway and MOST melanomas develop *de novo* (on their own, i.e. NOT from pre-existing nevus). Benign and dysplastic nevi are understood to, for the most part, arise independently of one another and most are acquired in young adult and teenage years (though, congenital nevi also exist).

The histological cartoon on the left is of a benign nevus and histological cartoon on right is of a malignant melanoma. There are three main histological features that distinguish them (as an oversimplification, dysplastic nevi fall somewhere in between): **1) Degree of cellular irregularity (atypia), 2) Degree of architectural disorder (location & configuration of melanocyte nests, 3) Degree of "stromal response" (i.e. inflammation).**

How can a benign entity could "breach" the basement membrane (e.g. notice the cluster of melanocytes in the dermis for the benign compound nevus on left; as a side note - they are blueish because of a phenomenon called the Tyndall effect, to be explained in a bit)? *Doesn't this go against the rule that a "hallmark" of invasive carcinoma (for many cell types, including epithelial cells and melanocytes) means invasion of the basement membrane as is the case for keratinocyte carcinoma!?* Well, here's a way to reconcile this: one theory is that acquired benign and dysplastic nevi result from aberrant migration of melanocytes (neural-crest derived cells) from below the epidermis to the dermal-epidermal (D-E) junction; it is *aberrant halted migration* (not invasion through basement membrane, per say) that may lead to presence of melanocytic clusters in dermis that form benign dermal nevi, for example. Malignant melanoma, in contrast, is believed to originate largely from atypical melanocytes at the D-E junction. Initially, atypical cells at D-E junction often undergo a so-called **horizontal growth phase** as they grow horizontally along the D-E junction. At this early stage, its usually curable by surgical excision since there is no or minimal invasion past basement membrane. However, unfortunately, melanoma spreads faster than SCC or BCC. At some point, it undergoes a **vertical growth phase** into the dermis (*invasion* through basement membrane – aha!) where it can spread into blood vessels, lymph nodes and other organs. **This is why prognosis is primarily determined by how "deep" tumor is. This is measured histologically as the Breslow thickness (from granular cell layer to deepest point of invasion of malignant cells).**

Authors: Jasmine Rana[1] BA & Arash Mostaghimi[2] MD, MPA, MPH
[1]Harvard Medical School: jasmine_rana@hms.harvard.edu
[2]Brigham and Women's Hospital, Harvard Medical School: amostaghimi@bwh.harvard.edu

DEFINITIONS

Benign nevi // Dysplastic nevi // Melanoma

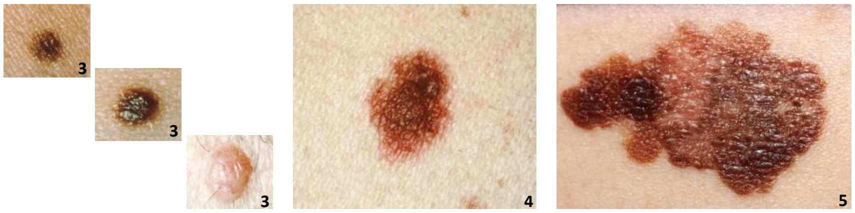

Images of benign nevi [3], dysplastic nevus [4], and malignant melanoma [5] from left to right. *Just by looking at the spectrum of lesions, you may have an intuition that some look more regular and some look more irregular!*

In far left image of benign nevi, from top to bottom: Junctional, compound, and dermal nevi, respectively, in which the melanocyte nests are in the D-E junction, D-E junction + dermis, and dermis, respectively. The deeper melanocytes get, the more elevated and less pigmented they get.

Authors: Jasmine Rana[1] BA & Arash Mostaghimi[2] MD, MPA, MPH
[1]Harvard Medical School: jasmine_rana@hms.harvard.edu
[2]Brigham and Women's Hospital, Harvard Medical School: amostaghimi@bwh.harvard.edu

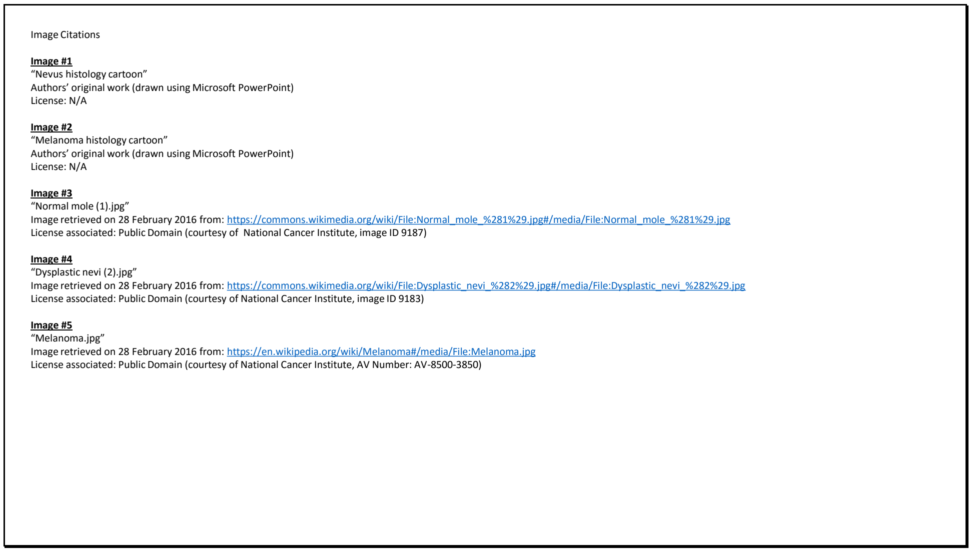

Image Citations

Image #1
"Nevus histology cartoon"
Authors' original work (drawn using Microsoft PowerPoint)
License: N/A

Image #2
"Melanoma histology cartoon"
Authors' original work (drawn using Microsoft PowerPoint)
License: N/A

Image #3
"Normal mole (1).jpg"
Image retrieved on 28 February 2016 from:
https://commons.wikimedia.org/wiki/File:Normal_mole_%281%29.jpg#/media/File:Normal_mole_%281%29.jpg
License associated: Public Domain (courtesy of National Cancer Institute, image ID 9187)

Image #4
"Dysplastic nevi (2).jpg"
Image retrieved on 28 February 2016 from:
https://commons.wikimedia.org/wiki/File:Dysplastic_nevi_%282%29.jpg#/media/File:Dysplastic_nevi_%282%29.jpg
License associated: Public Domain (courtesy of National Cancer Institute, image ID 9183)

Image #5
"Melanoma.jpg"
Image retrieved on 28 February 2016 from: https://en.wikipedia.org/wiki/Melanoma#/media/File:Melanoma.jpg
License associated: Public Domain (courtesy of National Cancer Institute, AV Number: AV-8500-3850)

"ABCDE" & Melanoma

Jasmine Rana[1], BA

Arash Mostaghimi[2], MD, MPA, MPH

[1]Harvard Medical School: jasmine_rana@hms.harvard.edu

[2]Brigham and Women's Hospital, Harvard Medical School: amostaghimi@bwh.harvard.edu

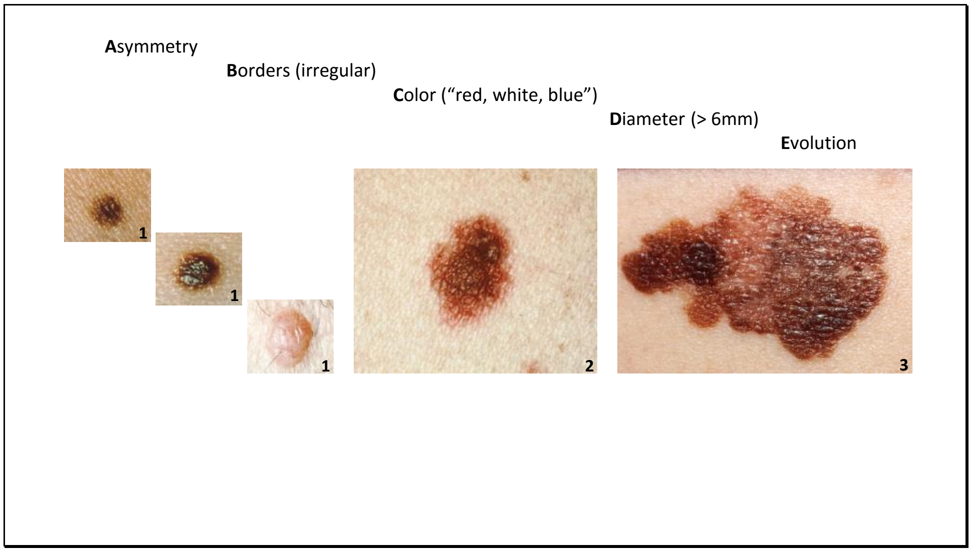

Primary care doctors, dermatologists, and patients often use the "ABCDE rule" (mnemonic listed above) to distinguish dysplastic nevi/melanoma from benign nevi*. Typically, one or more of these signs in a patient at risk (e.g. fair skin/family history of melanoma/risk factors for melanoma, e.g. tanning beds) may warrant referral to a dermatologist and/or possible biopsy. See this JAMA article for sensitivity and specificity of each of the ABCDE factors:

> Abbasi NR, Shaw HM, Rigel DS, et al. Early diagnosis of cutaneous melanoma: Revisiting the abcd criteria. *JAMA*. 2004;292(22): 2771-2776.

Asymmetry & Borders: Contrast the benign nevi with dysplastic nevus/melanoma in terms of symmetry and border irregularity.

Colors to be on the lookout for:
- Red – from bleeding/inflammation
- White – from **regression** (i.e. increased production of collagen and replacement of areas of the tumor with fibrosis (scarring) due to immune activation against the "foreign" tumor cells)
- Blue – from melanin deep in dermis; this is due to the *Tyndall effect*: preferential absorption of long wavelengths of light by melanin deeper in the dermis and scattering of shorter wavelengths (i.e. the blue end of the spectrum) by collagen bundles in the dermis (accordingly, melanin in upper epidermis above the collagen bundles in dermis appears the most pigmented).

Also look for general brown-black color variation *within* a lesion.

Diameter: Note that 6 mm is about the size of a pencil eraser!

Evolution: Note that "E" (evolution of lesion) usually comes from asking patient ("Has the lesion gotten bigger? Does it hurt? Bleeding? Itching?) and/or watching the lesion over time.

In addition to ABCDE features, there is another helpful clinical tool to identify worrisome pigmented lesions called the **"ugly duckling sign".** The idea is this: if you have a patient who makes a lot of moles (which may appear normal or even a little dysplastic), you don't want to biopsy and/or remove every single one because as long as the patient doesn't have a genetic disorder/underlying risk factor making them more predisposed to melanoma, most lesions will not have malignant potential. In general, pigmented lesions with malignant potential look slightly different than the normal "flavor" of moles a particular individual makes. Thus, the goal is to find the lesion(s) that are "ugly duckling(s)" or the odd ones out.

**Why is this important? Early detection of early melanomas or dysplastic nevi may prompt excision of these lesions, which can prevent or halt tumor progression. However, we wouldn't want to excise every single pigmented lesion we see! ABCDE features help us identify the pigmented lesions that deserve a second-look and possible biopsy and/or removal.*

Authors: Jasmine Rana[1] BA & Arash Mostaghimi[2] MD, MPA, MPH
[1]Harvard Medical School: jasmine_rana@hms.harvard.edu
[2]Brigham and Women's Hospital, Harvard Medical School: amostaghimi@bwh.harvard.edu

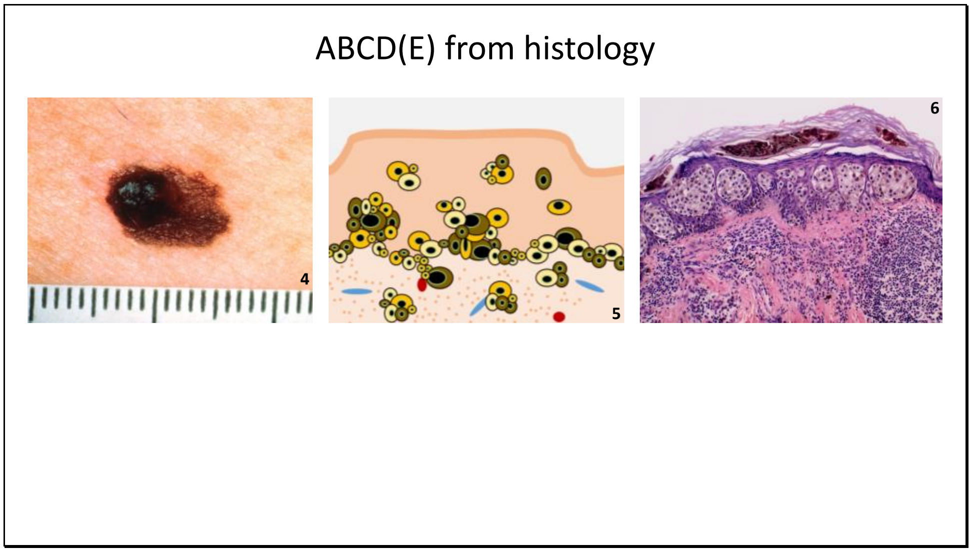

While previously discussed in reference to clinical appearance of lesions, ABCD(E*) features have a basis in what is going histologically!

Starting on left with a nodular melanoma, note: the asymmetry, irregular borders, 1 cm size, and color variation. In the middle is a 2D histological cartoon image showing what's going on "under the hood" – note the atypical melanocytes invading from D-E junction into dermis in irregular cluster patterns → asymmetry and border irregularity seen clinically. In the H&E stain at the far right, also notice the atypical melanocytes produce differing amounts of pigment (shown as brown whirls in cells) → color variation seen clinically.

**E is in parentheses because, as mentioned earlier, evidence of the lesion's evolution comes from asking the patient and/or watching the lesion over time.*

Authors: Jasmine Rana[1] BA & Arash Mostaghimi[2] MD, MPA, MPH
[1]Harvard Medical School: jasmine_rana@hms.harvard.edu
[2]Brigham and Women's Hospital, Harvard Medical School: amostaghimi@bwh.harvard.edu

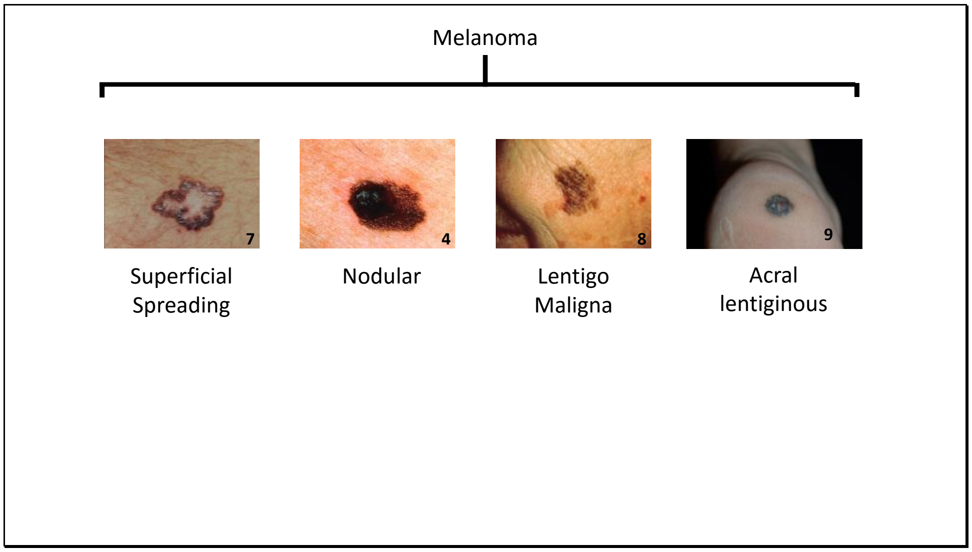

Major Subtypes of Melanoma (from left to right):

Superficial spreading: Most common (60-70% of all melanomas). Usually a spreading pigmented plaque with irregular borders; tend to have a good prognosis due to an early horizontal growth phase.

Nodular: Often rapid growth and more aggressive because of early vertical growth phase and often non-existent horizontal growth phase.

Lentigo* Maligna: Commonly macular pigmented lesions, most common in elderly. Often insidious with horizontal phase early on, but malignant cells can often extend beyond the clinical lesion, often making excision difficult.

Acral lentiginous*: On hands/feet/fingers/toes/nails. Often involves thumb, can be confused with hematoma. Higher percentage of melanomas are acral in Asians and African Americans compared to other populations.

As a side note, the subtypes of melanoma are defined from their clinical and histological features, but don't have any significant prognostic value. The most important histological prognostic marker (regardless of subtype) is the Breslow thickness.

**These terms are used because of their visual similarity to a lentigo, which is a benign pigmented lesion distinct from moles.*

Authors: Jasmine Rana[1] BA & Arash Mostaghimi[2] MD, MPA, MPH
[1]Harvard Medical School: jasmine_rana@hms.harvard.edu
[2]Brigham and Women's Hospital, Harvard Medical School: amostaghimi@bwh.harvard.edu

Image Citations

Image #1
"Normal mole (1).jpg"
Image retrieved on 28 February 2016 from: https://commons.wikimedia.org/wiki/File:Normal_mole_%281%29.jpg#/media/File:Normal_mole_%281%29.jpg
License associated: N/A Public Domain (courtesy of National Cancer Institute, image ID 9187)

Image #2
"Dysplastic nevi (2).jpg"
Image retrieved on 28 February 2016 from: https://commons.wikimedia.org/wiki/File:Dysplastic_nevi_%282%29.jpg#/media/File:Dysplastic_nevi_%282%29.jpg
License associated: N/A Public Domain (courtesy of National Cancer Institute, image ID 9183)

Image #3
"Melanoma.jpg"
Image retrieved on 28 February 2016 from: https://en.wikipedia.org/wiki/Melanoma#/media/File:Melanoma.jpg
License associated: N/A Public Domain (courtesy of National Cancer Institute, AV Number: AV-8500-3850)

Image #4
"Melanoma Asymmetry.jpg"
Image retrieved on 28 February 2016 from: https://en.wikipedia.org/wiki/File:Melanoma_Asymmetry.jpg#/media/File:Melanoma_Asymmetry.jpg
License associated: N/A Public Domain (courtesy of National Cancer Institute, AV Number: AV-8809-4035)

Image #5
"Melanoma histology cartoon"
Authors' original work (drawn using Microsoft PowerPoint)
License: N/A

Image #6
"Skin Tumors-450.jpg"
Image retrieved on 28 February 2016 from: https://en.wikipedia.org/wiki/Superficial_spreading_melanoma#/media/File:Skin_Tumors-450.jpg
License associated: http://creativecommons.org/licenses/by-sa/3.0/

Image Citations

Image #1
"Normal mole (1).jpg"
Image retrieved on 28 February 2016 from:
https://commons.wikimedia.org/wiki/File:Normal_mole_%281%29.jpg#/media/File:Normal_mole_%281%29.jpg
License associated: Public Domain (courtesy of National Cancer Institute, image ID 9187)

Image #2
"Dysplastic nevi (2).jpg"
Image retrieved on 28 February 2016 from:
https://commons.wikimedia.org/wiki/File:Dysplastic_nevi_%282%29.jpg#/media/File:Dysplastic_nevi_%282%29.jpg
License associated: Public Domain (courtesy of National Cancer Institute, image ID 9183)

Image #3
"Melanoma.jpg"
Image retrieved on 28 February 2016 from: https://en.wikipedia.org/wiki/Melanoma#/media/File:Melanoma.jpg
License associated: Public Domain (courtesy of National Cancer Institute, AV Number: AV-8500-3850)

Image #4
"Melanoma Asymmetry.jpg"
Image retrieved on 28 February 2016 from:
https://en.wikipedia.org/wiki/File:Melanoma_Asymmetry.jpg#/media/File:Melanoma_Asymmetry.jpg
License associated: Public Domain (courtesy of National Cancer Institute, AV Number: AV-8809-4035)

Image #5
"Melanoma histology cartoon"
Authors' original work (drawn using Microsoft PowerPoint)
License: N/A

Image #6
"Skin Tumors-450.jpg"
Image retrieved on 28 February 2016 from:
https://en.wikipedia.org/wiki/Superficial_spreading_melanoma#/media/File:Skin_Tumors-450.jpg
License associated: http://creativecommons.org/licenses/by-sa/3.0/

Authors: Jasmine Rana[1] BA & Arash Mostaghimi[2] MD, MPA, MPH
[1]Harvard Medical School: jasmine_rana@hms.harvard.edu
[2]Brigham and Women's Hospital, Harvard Medical School: amostaghimi@bwh.harvard.edu

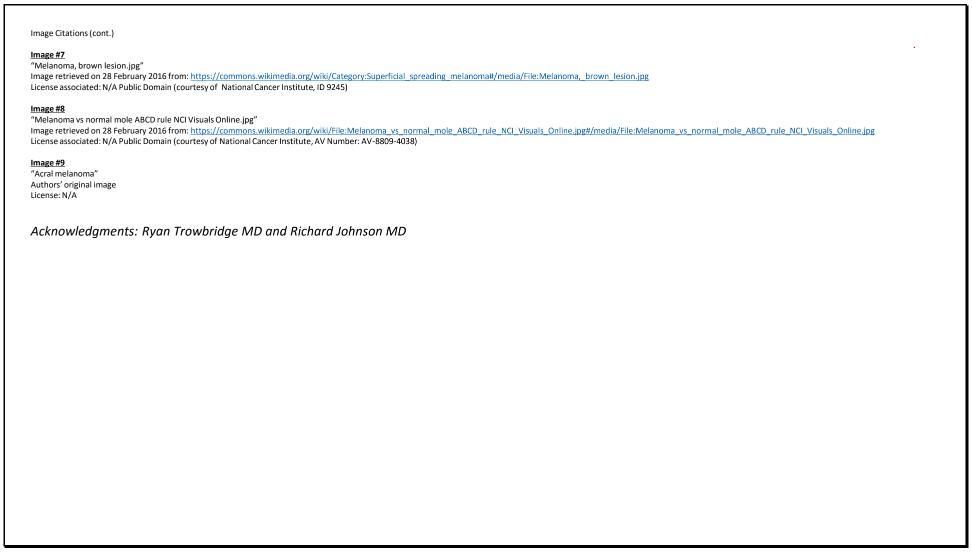

Image Citations (cont.)

Image #7
"Melanoma, brown lesion.jpg"
Image retrieved on 28 February 2016 from:
https://commons.wikimedia.org/wiki/Category:Superficial_spreading_melanoma#/media/File:Melanoma,_brown_lesion.jpg
License associated: Public Domain (courtesy of National Cancer Institute, ID 9245)

Image #8
"Melanoma vs normal mole ABCD rule NCI Visuals Online.jpg"
Image retrieved on 28 February 2016 from:
https://commons.wikimedia.org/wiki/File:Melanoma_vs_normal_mole_ABCD_rule_NCI_Visuals_Online.jpg#/media/File:Melanoma_vs_normal_mole_ABCD_rule_NCI_Visuals_Online.jpg
License associated: Public Domain (courtesy of National Cancer Institute, AV Number: AV-8809-4038)

Image #9
"Acral melanoma"
Authors' original image
License: N/A

Acknowledgments: Ryan Trowbridge MD and Richard Johnson MD
