# Supplementary material for: Introduction to Skin Cancer: A Video Module
Source: MedEdPORTAL. 2016 Aug 5;12:10431. doi: 10.15766/mep_2374-8265.10431 (PMC6464471; doi:10.15766/mep_2374-8265.10431)
Supplement: Supplementary file 1 — A. Skin Cancer Learner Guide.pdf B. Video 1- Intro to Skin Cancer.mp4 C. Video 2- Keratinocyte Skin Cancer.mp4 D. Video 3- Overview of Pigmented Lesions.mp4 E. Video 4- ABCDE and Melanoma.mp4 F. Skin Cancer Annotated Slides.pdf G. Skin Cancer Self-Assessment.pdf [file mep-12-10431-s001.zip › G. Skin Cancer Self-Assessment.pdf]

Authors: Jasmine Rana[1] BA & Arash Mostaghimi[2] MD, MPA, MPH
[1]Harvard Medical School: jasmine_rana@hms.harvard.edu
[2]Brigham and Women's Hospital, Harvard Medical School: amostaghimi@bwh.harvard.edu

Introduction to Skin Cancer: A Video Module

Review Questions

1. What is the correct order of prevalence for the most common skin cancers?
    a. BCC > SCC > Melanoma
    b. SCC > BCC > Melanoma
    c. SCC > Melanoma > BCC
    d. BCC > Melanoma > SCC
    e. Melanoma > BCC > SCC

2. What is the correct order of mortality caused by the most common skin cancers?
    a. BCC > SCC > Melanoma
    b. SCC > BCC > Melanoma
    c. Melanoma > SCC > BCC
    d. SCC > Melanoma > BCC
    e. BCC > Melanoma > SCC

3. Invasive SCC and BCC on histology both show all of the following features EXCEPT:
    a. Cellular atypia
    b. Pleomorphic nuclei
    c. Dermal inflammatory infiltrate
    d. Invasion of epidermal cells past the basement membrane
    e. Peripheral palisading

4. What is the most accurate histological description of the lesion?

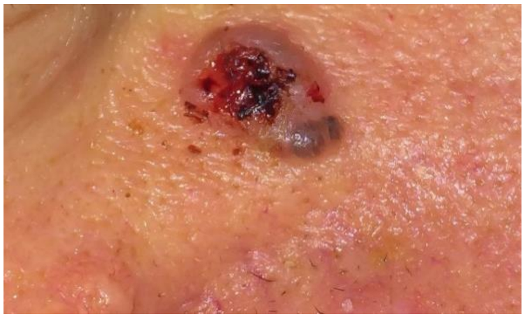

Authors' original image
(Acknowledgment: Ryan Trowbridge MD)

Authors: Jasmine Rana[1] BA & Arash Mostaghimi[2] MD, MPA, MPH
[1]Harvard Medical School: jasmine_rana@hms.harvard.edu
[2]Brigham and Women's Hospital, Harvard Medical School: amostaghimi@bwh.harvard.edu

a. Basaloid nests with retraction artifacts invading into the dermis
b. Atypical epidermal cells invading into the dermis with keratin pearls
c. Atypical nevus cells invading into the dermis
d. Acanthosis and cellular epidermal atypia confined to the epidermis
e. Well-demarcated clusters of nevus cells in the dermis

5. What is the most accurate histological description of the lesion?

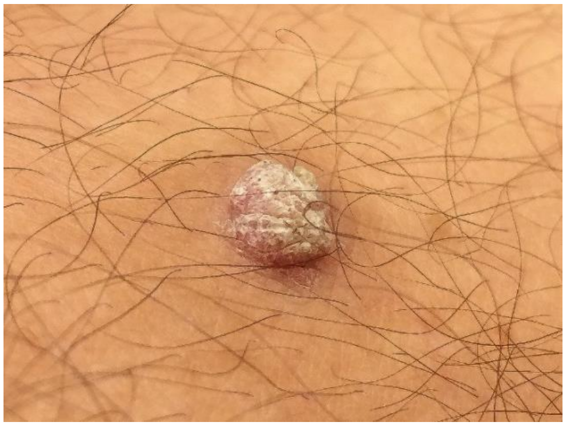

Authors' original image
(Acknowledgment: Ryan Trowbridge MD)

a. Basaloid nests with retraction artifacts invading into the dermis
b. Atypical epidermal cells invading into the dermis with keratin pearls
c. Atypical nevus cells invading into the dermis
d. Acanthosis and cellular epidermal atypia confined to the epidermis
e. Well-demarcated clusters of nevus cells in the dermis

6. *True or False:* Having dysplastic nevi increases your risk for melanoma because most dysplastic nevi eventually turn into melanoma.

7. *True or False:* Early melanomas generally have all of the ABCDE criteria.

8. *True or False:* Benign dermal nevi can appear blue.

9. What is the most important histological prognostic factor for this skin cancer?

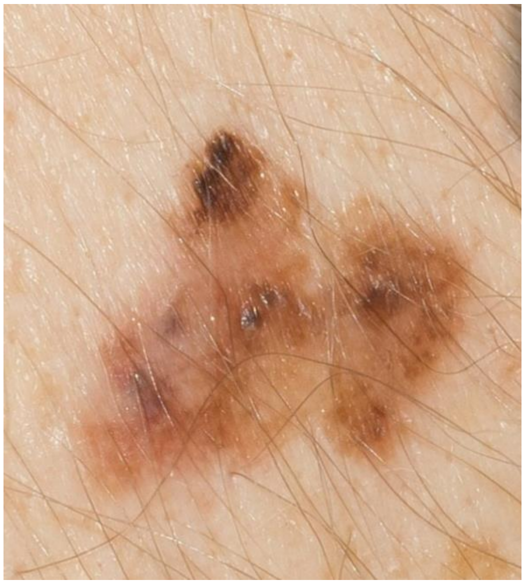

Authors' original image
(Acknowledgment: Ryan Trowbridge MD)

a. Mitotic rate
b. Degree of ulceration
c. Presence of regression
d. Breslow thickness
e. Diameter of lesion

10. The phenomenon of "regression" in some melanomas is due to:
    a. Scarring in response to external trauma
    b. Decreased production of collagen in the dermis
    c. Immune activation against neoplastic melanocytes
    d. Production of white pigment by atypical melanocytes
    e. High mitotic rate of atypical melanocytes

Authors: Jasmine Rana[1] BA & Arash Mostaghimi[2] MD, MPA, MPH
[1]Harvard Medical School: jasmine_rana@hms.harvard.edu
[2]Brigham and Women's Hospital, Harvard Medical School: amostaghimi@bwh.harvard.edu

Answers/Explanations

1. ***Answer: a.*** *Note that mortality due to skin cancer is the opposite: Melanoma > SCC > BCC.*
2. ***Answer: c.*** *Note that prevalence of skin cancer is the opposite: BCC > SCC > Melanoma.*
3. ***Answer: e.*** *Peripheral palisading is unique to BCC and describes the histological phenomenon of basaloid cells that neatly line up along the periphery of cancerous basaloid nests. Common features of keratinocyte skin cancers on histology include:*
   - *Cellular atypia & pleomorphic nuclei: signs of uncontrolled hyperproliferation of cells.*
   - *Dermal inflammatory infiltrate: the body's immune system is activated and trying to fight the tumor.*
   - *Invasion of epidermal cells past the basement membrane: what defines both of these as not being superficial (in situ) tumors relegated to the epidermis but true invasive malignancies.*
4. ***Answer: a.*** *Correct diagnosis: BCC. Note rolled borders and central ulceration. The other descriptions may commonly describe: B) SCC, C) Melanoma or dysplastic nevus, D) Actinic keratosis, E) Lentigo or benign nevi.*
5. ***Answer: b.*** *Correct diagnosis: SCC. Note hyperkeratotic (scaly) surface. The other descriptions may commonly describe: A) BCC, C) melanoma, D) SCC in situ, E) Benign dermal nevus.*
6. ***False*** *– while dysplastic nevi ARE a phenotypic marker for increased melanoma risk, only ~1/3 of melanoma develop from pre-existing nevi, most occur de nevo. Dysplastic nevi are epidemiologically a marker for increased risk of melanoma but the pathophysiology of this connection is unclear.*
7. ***False*** *– there is no exact "threshold" for performing a biopsy of a suspicious pigmented lesion, but rather, take into account the patient's clinical risk factors, story (assess evolution), and presence of ANY of the ABCD features – it is an art as much of a science! In addition to having a trained eye, a dermatoscope, (a magnifying glass that shines polarized light) can also be useful in assessing suspicious vs. benign pigment patterns in the lesion.*
8. ***True*** *– blue color simply reflects that melanin is located deep in the dermis. A homogenous blue color in a well-demarcated dermal nevus is not generally worrisome. However, blue patchy spots in a pigmented lesion that has other ABCDE features is worrisome for a dysplastic nevus and/or melanoma.*
9. ***Answer: d.*** *Breslow thickness is the most important histological prognostic factor for melanoma. It is measured from the granular layer of the epidermis to the deepest point of invasion. Ulceration and mitotic rate are additional histological prognostic factors. Regression is not an independent indicator of prognosis.*
10. ***Answer: c.*** *Regression involves increased production of collagen and replacement of areas of the tumor with fibrosis (scarring) due to immune activation against the "foreign" tumor cells. There is conflicting data on prognostic value of regression in melanomas.*
