# Supplementary material for: Introduction to Skin Cancer: A Video Module
Source: MedEdPORTAL. 2016 Aug 5;12:10431. doi: 10.15766/mep_2374-8265.10431 (PMC6464471; doi:10.15766/mep_2374-8265.10431)
Supplement: Supplementary file 1 — A. Skin Cancer Learner Guide.pdf B. Video 1- Intro to Skin Cancer.mp4 C. Video 2- Keratinocyte Skin Cancer.mp4 D. Video 3- Overview of Pigmented Lesions.mp4 E. Video 4- ABCDE and Melanoma.mp4 F. Skin Cancer Annotated Slides.pdf G. Skin Cancer Self-Assessment.pdf [file mep-12-10431-s001.zip › _Educational Summary Report_10431.pdf]

MedEdPORTAL®
The Journal of Teaching and Learning Resources

Original Publication

OPEN ACCESS

Introduction to Skin Cancer: A Video Module

Jasmine Rana*, Arash Mostaghimi, MD, MPA, MPH
*Corresponding author: jasmine_rana@hms.harvard.edu

Citation: Rana J, Mostaghimi A. Introduction to skin cancer: a video module. *MedEdPORTAL*. 2016;12:10431. https://doi.org/10.15766/mep_2374-8265.10431

Copyright: © 2016 Rana and Mostaghimi. This is an open-access article distributed under the terms of the Creative Commons Attribution license.

Abstract

Introduction: This introductory skin cancer video module engages novice health care professionals to make histological-clinical correlations for the three most common skin cancers: basal cell carcinoma (BCC), squamous cell carcinoma (SCC), and melanoma. The goal of this video module is to engage novice health care professionals (e.g., allied health, medical, nursing students) to go beyond the all-too-common phenomenon of memorizing gross and histological features of skin cancers without reference to the relationship between these features. **Methods:** By explicitly highlighting underlying histological-clinical correlations for BCC, SCC, and melanoma, this video module helps learners build a deeper and lasting knowledge of these common diseases. Materials in this module include a learner guide with instructions on how to complete the module; four chalk-talk videos that discuss classification of skin cancers, histological-clinical correlations for BCC, SCC, and melanoma, and how to distinguish among benign nevi, dysplastic nevi, and melanoma (total viewing time 54 minutes, 22 seconds); annotated slides used in the videos; and 10 multiple-choice self-assessment questions. **Results:** This module was successfully incorporated into the first-year flipped classroom curriculum for medical and dental students at Harvard Medical School. Written comments from students revealed that they enjoyed watching short concept videos to prepare for in-class, case-based discussions of BCC, SCC, and melanoma. **Discussion:** By illustrating histological-clinical correlations and reducing cognitive load of the material through use of cartoons and prototypical clinical images, this video module is an accessible initial resource for an emerging generation of millennial health care professionals to learn about common skin cancers.

Keywords

Editor's Choice, Skin Neoplasms, Melanoma, ABCDE, Skin Cancer, Nevus, Nevi, Basal Cell, Epithelial Cells, Squamous Cell, NMSC

Appendices

A. Skin Cancer Learner Guide .pdf
B. Video 1- Intro to Skin Cancer.mp4
C. Video 2- Keratinocyte Skin Cancer.mp4
D. Video 3- Overview of Pigmented Lesions.mp4
E. Video 4- ABCDE and Melanoma.mp4
F. Skin Cancer Annotated Slides.pdf
G. Skin Cancer Self-Assessment.pdf

All appendices are peer reviewed as integral parts of the Original Publication.

Educational Objectives

By the end of this module, learners will be able to:

1. Describe how the most common types of skin cancer (squamous cell carcinoma [SCC], basal cell carcinoma [BCC], and melanoma) are classified.
2. Understand the relationship between hallmark histological and clinical features for SCC, BCC, and melanoma.
3. Describe key histological differences (architectural, cellular, and stromal) between nevi and melanoma.
4. Identify clinical ABCDE (asymmetry, border irregularity, color variation, diameter > 6 mm, and evolution) features of dysplastic nevi and melanoma that make them distinct from benign nevi.
5. Describe the four main subtypes of melanoma (superficial, nodular, lentigo maligna, and acral).
6. Describe the three main subtypes of benign nevi (junctional, compound, and dermal).

Introduction

The flipped classroom is an emerging pedagogy being adapted by medical schools across the country to encourage more effective self-directed learning, especially in preclinical years.[1] This video module was

produced for a new flipped classroom dermatology curriculum for 170 first-year medical (135) and dental (35) students at Harvard Medical School (HMS), first implemented in Fall 2015 (Harvard medical and dental students enroll in the same 14-month preclinical curriculum).

Learner gains (via knowledge and attitude outcomes) have been previously documented when using concept videos in the context of a flipped classroom in health professions education.[2-4] Moreover, concept videos and the overall flipped classroom pedagogy are attractive to millennials, who are visual learners and prefer to learn at their own pace.[5,6]

Because they require baseline knowledge of clinical and histological terms and more familiarity with the clinical context, readily available self-directed introductory learning resources about skin cancer[7-9] are not developmentally appropriate for first-year medical students. This is not surprising given that most preclinical medical students have limited exposure to dermatology.[10] Many introductory dermatological texts target senior medical students and/or dermatology residents. To begin to fill the gap for novice learners, we created a skin cancer video module at our institution for first-year medical and dental students to view as a primary resource in preparation for case-based learning activities facilitated by faculty in the classroom. The video format also has the benefit of being learned any time, any place, thus allowing students with little to no formal coverage of dermatology in early years of any health professions track to supplement their education with this self-directed module.

In addition to using dynamic visual displays that engage millennial learners, the video module was designed using cognitive load theory.[11] For example, side-by-side comparisons of cartoon drawings and actual hematoxylin and eosin stain histological images help reduce the cognitive load for novice learners as they activate prior knowledge to build and reorganize schemas for understanding the key histological features of skin cancers. The video module was also designed using the learning science principle of interleaving.[12] Although the four videos have distinct learning goals, there is purposeful interleaving of concepts related to three cancers—basal cell carcinoma (BCC), squamous cell carcinoma (SCC), and melanoma—within and among videos to emphasize causal pathways (e.g., atypical cancer cells can stimulate an immune response) common to all of the skin cancers and facilitate long-term retention of salient distinctions among these lesions.

This resource is most appropriate for novice health care professionals. Only prior knowledge of the basic structure of the skin (i.e., layers of epidermis, dermis, subcutaneous fat and sublayers of the epidermis) and how to describe dermatological lesions (e.g., using terms like papule, plaque, macule, etc.) is required prior to viewing these videos; this material is typically covered at the start of most preclinical dermatology curricula. It is also helpful to have an understanding of core concepts in histology, but many key concepts can be inferred from the videos. Finally, although these videos were designed in the context of a flipped classroom model, they can be readily adapted for use in a diverse range of settings (e.g., viewed by learners prior to or after a traditional lecture, viewed to supplement clinical encounters, viewed when the learner wants to review fundamental concepts, etc.).

Methods

Given that many health professions schools aim to foster self-directed learning in time-constrained curricula, this module is designed for students to complete in 1.5 to 2 hours. Although no direct faculty support is required for learners to complete this freestanding module, we envision the module will supplement subsequent learning experiences with faculty in the classroom or clinic where students can expand on content presented here.

We provided this module to first-year medical and dental students in an introductory dermatology curriculum, but the target audience can be any novice health professions student. In a flipped classroom design, students were assigned to view these videos and complete self-assessment questions prior to faculty-led sessions that involved discussion of clinical cases involving BCC, SCC, and melanoma (approximately 1 hour each).

Logistically, all that is required for learners to complete this module is a computing device that can play MP4 videos and support viewing PDF files. Learning materials in the module are listed below in the recommended learning sequence:

- Appendix A. Skin Cancer Learner Guide contains a session overview, learning goals, and list of key terms.
- Appendix B. Video 1-Intro to Skin Cancer includes classification of SCC, BCC, and melanoma (8 minutes, 15 seconds).
- Appendix C. Video 2-Keratinocyte Skin Cancer includes the histological-clinical correlations of BCC and SCC (18 minutes, 37 seconds).
- Appendix D. Video 3-Overview of Pigmented Lesions includes the histological and clinical features of nevi and melanoma (13 minutes, 56 seconds).
- Appendix E. Video 4-ABCDE and Melanoma includes the ABCDE (asymmetry, border irregularity, color variation, diameter > 6 mm, and evolution) rule and clinical-histological correlations (13 minutes, 34 seconds).
- Appendix F. Skin Cancer Annotated Slides contains the slides featured in the videos.
- Appendix G. Skin Cancer Self-Assessment contains 10 multiple-choice questions, with answers and explanations provided.

Note that videos should be viewed in sequence and self-assessment questions should be completed at the end of the module. If learners choose not to watch all the videos in one sitting, they may find it useful to view Videos 1 and 2 together (total viewing time is 26 minutes, 52 seconds) followed by Videos 3 and 4 (total viewing time is 27 minutes, 30 seconds) as these video pairs roughly cover distinct topics (i.e., keratinocyte skin cancer and melanoma/nevi, respectively). Self-assessment questions were ungraded in order to create a safe learning space, and answers were provided to give learners real-time feedback with clinical pearls. Annotated slides were offered as another resource to help synthesize the content upon student request based on feedback from prior courses.

Although these videos can be viewed by students independent of formal coursework, from our experience, they work well when paired with a clinical or classroom-based activity that can activate prior knowledge students have learned by watching the videos. Videos were created using Microsoft PowerPoint 2016 (slides), Wacom Bamboo Tablet model CTL-470 (video annotations), Blue Microphones Snowball USB Microphone (sound), and TechSmith Camtasia Studio Version 8.6 (recording video and editing).

Results

One-hundred seventy first-year medical and dental students at HMS were assigned the video module to prepare for in-class activities led by individual faculty members. Although we could not quantify what proportion of students viewed the videos, comments from students about the videos in an end-of-course survey were overwhelmingly positive, with many citing the module as one of the most effective preparatory materials for the course. Below are representative excerpts from student comments about the materials included in the module:

- “The Skin Cancer module prep materials were extremely effective. . . . For instance, I watched the videos, and then used the annotated slides to review the material later or during the [review questions].”
- “I really appreciate the use of cartoon parallels to histological slides as a priming for understanding the actual histological slides.”
- “I will never forget how to diagnose skin cancer because of the clarity in which the material was explained. The technology was appropriately used and the information was extremely well represented.”
- “The videos were extremely well done because they were relatively short, they were informative, and they had a great mix of real images and slides.”

- “The videos were so straightforward and did a phenomenal job connecting the histology with the gross presentation of the lesions.”
- “[T]here was just enough repetition so I felt like I recalled the information after completing the prep.”

Discussion

Too often, gross and histological manifestations of skin cancer are just memorized by medical students without deep appreciation for the underlying clinical-histological correlations. The goal of this introductory skin cancer video module was to create a developmentally appropriate, engaging, video-based resource for novice learners that can serve as the foundation for further learning about these cancers in clinical or classroom-based settings. The video module can be used in a wide variety of contexts but has worked particularly well in a flipped classroom for a preclinical joint medical-dental school dermatology block.

The greatest challenge was the time and effort involved in producing the concept videos. This required choosing and formatting original and/or noncopyrighted images in a palatable and engaging format. We found it was beneficial to have a fourth-year medical student produce and record the videos because she had more cognitive and social congruence with first-year medical students than did experienced faculty members, which is consistent with literature on near-peer teaching,[13] making the material more accessible to these learners as evidenced by written feedback comments.

This resource was not meant to be comprehensive, but the major limitation of the resource is that it does not contain in-depth content about epidemiology, risk factors, and management of BCC, SCC, melanoma, or nevi. This information can be supplemented through textbook readings and/or taught at later stages of training.

In the future, our goal is to create similar videos for histological-clinical correlations of other common skin lesions. A disadvantage of most dermatology texts is that static histological and clinical images are often not annotated and are difficult to interpret by novice learners. A video offers a unique medium to highlight histological-clinical correlations that are often inaccessible or not very well highlighted for novice learners in textbooks.

Given that students appreciate multiple learning modalities, which is consistent with Kolb’s theory for experiential adult learning[14] and data on millennial learners,[5] more kinesthetic and interactive features (e.g., flash cards of lesions that allow students to directly circle pathologies and highlight the hallmark histological findings) may be valuable supplements to these concept videos in the future. We also hope to do more quantitative outcome studies (e.g., pre- and posttest) to evaluate the effectiveness of this module on learner knowledge outcomes in the future.

Jasmine Rana: MD-MMSc Candidate, Harvard Medical School

Arash Mostaghimi, MD, MPA, MPH: Assistant Professor of Dermatology and Internal Medicine, Harvard Medical School; Director of Dermatology Inpatient Services and Co-Director of the Complex Medical Dermatology Fellowship Program, Brigham and Women’s Hospital

---

Disclosures
None to report.

Funding/Support
None to report.

Ethical Approval
Reported as not applicable.

References

1. Prober CG, Heath C. Lecture halls without lectures—a proposal for medical education. *N Engl J Med.* 2012;366(18):1657-1659. http://dx.doi.org/10.1056/NEJMp1202451

2. Khanova J, Roth MT, Rodgers JE, McLaughlin JE. Student experiences across multiple flipped courses in a single curriculum. *Med Educ*. 2015;49(10):1038-1048. http://dx.doi.org/10.1111/medu.12807
3. McLaughlin JE, Roth MT, Glatt DM, et al. The flipped classroom: a course redesign to foster learning and engagement in a health professions school. *Acad Med*. 2014;89(2):236-243. http://dx.doi.org/10.1097/ACM.0000000000000086
4. Morgan H, McLean K, Chapman C, Fitzgerald J, Yousuf A, Hammoud M. The flipped classroom for medical students. *Clin Teach*. 2015;12(3):155-160. http://dx.doi.org/10.1111/tct.12328
5. Matulich E, Papp R, Haytko DL. Continuous improvement through teaching innovations: a requirement for today's learners. *Marketing Educ Rev*. 2008;18(1):1-7. http://dx.doi.org/10.1080/10528008.2008.11489017
6. Phillips CR, Trainor JE. Millennial students and the flipped classroom. In: Proceedings of the American Society of Business and Behavioral Sciences; February 20-23, 2014; Las Vegas, NV.
7. Basic dermatology curriculum. American Academy of Dermatology Web site.https://www.aad.org/education/basic-derm-curriculum. Accessed January 31, 2016.
8. Nguyen L, Mahalingam M, Garg A. Dermatology clinical case modules: 70-year-old man with a red crusty bump on his right arm. *MedEdPORTAL Publications*. 2010;6:8055. http://dx.doi.org/10.15766/mep_2374-8265.8055
9. Nguyen L, Mahalingam M, Garg A. Dermatology clinical case modules: 40-year-old woman with a dark mole. *MedEdPORTAL Publications*. 2010;6:8067. http://dx.doi.org/10.15766/mep_2374-8265.8067
10. McCleskey PE, Gilson RT, DeVillez RL. Medical Student Core Curriculum in Dermatology Survey. *J Am Acad Dermatol*. 2009;61(1):30-35. http://dx.doi.org/10.1016/j.jaad.2008.10.066
11. Young JQ, Van Merrienboer J, Durning S, Ten Cate O. Cognitive load theory: implications for medical education: AMEE Guide No. 86. *Med Teach*. 2014;36(5):371-384. http://dx.doi.org/10.3109/0142159X.2014.889290
12. Brown, PC, Roediger HL III, McDaniel, MA. *Make It Stick: The Science of Successful Learning.* Cambridge, MA: Belknap Press; 2014.
13. Lockspeiser TM, O'Sullivan P, Teherani A, Muller J. Understanding the experience of being taught by peers: the value of social and cognitive congruence. *Adv Health Sci Educ Theory Pract*. 2008;13(3):361-372. http://dx.doi.org/10.1007/s10459-006-9049-8
14. Armstrong E, Parsa-Parsi R. How can physicians' learning styles drive educational planning? *Acad Med*. 2005;80(7):680-684. http://dx.doi.org/10.1097/00001888-200507000-00013

Received: April 8, 2016 | **Accepted:** June 23, 2016 | **Published:** August 5, 2016
