# Supplementary material for: Introduction to Skin Cancer: A Video Module
Source: MedEdPORTAL. 2016 Aug 5;12:10431. doi: 10.15766/mep_2374-8265.10431 (PMC6464471; doi:10.15766/mep_2374-8265.10431)
Supplement: Supplementary file 1 — A. Skin Cancer Learner Guide.pdf B. Video 1- Intro to Skin Cancer.mp4 C. Video 2- Keratinocyte Skin Cancer.mp4 D. Video 3- Overview of Pigmented Lesions.mp4 E. Video 4- ABCDE and Melanoma.mp4 F. Skin Cancer Annotated Slides.pdf G. Skin Cancer Self-Assessment.pdf [file mep-12-10431-s001.zip › A. Skin Cancer Learner Guide.pdf]

Authors: Jasmine Rana[1] BA & Arash Mostaghimi[2] MD, MPA, MPH
[1]Harvard Medical School: jasmine_rana@hms.harvard.edu
[2]Brigham and Women's Hospital, Harvard Medical School: amostaghimi@bwh.harvard.edu

Introduction to Skin Cancer: A Video Module
Learner Guide

Summary

Although skin cancer does not make it into the list of the top 3 "most common" cancers in the US (breast/prostate, lung, colon), keratinocyte-derived skin cancers (basal and squamous cell carcinoma) are actually the world's most common cancers! They are not included in the "top 3" list because, if caught early, as most are, they are essentially curable. On the other hand, melanoma, the third most common skin cancer, often has a less favorable prognosis than keratinocyte-derived skin cancers. Although some early melanoma lesions are also essentially curable if caught early, as a whole, melanoma accounts for < 2% of all skin cancer, but accounts for approximately 80% deaths from skin cancer.

The primary goal of this video module is to give you knowledge and tools to confidently and accurately identify the 3 most common skin cancers on your patients. Dermatologists are usually not the first clinicians to identify skin cancer. Simply being aware of what to look for (and, in particular for melanoma, being able to distinguish a malignant pigmented lesion from a benign pigmented lesion) can save lives in whatever specialty you enter. By the end of this 4-video module (total viewing time = 54:22 min), learners will be able to *(videos with specific learning goals are **bolded**)*:

1. *Describe how the most common types of skin cancer (squamous cell carcinoma (SCC), basal cell carcinoma (BCC), and melanoma) are classified* ***(Video 1)***
2. *Understand the relationship between hallmark histological and clinical features for:*
    a. *SCC* ***(Video 2)***
    b. *BCC* ***(Video 2)***
    c. *Melanoma* ***(Video 4)***
3. *Describe key histological differences (architectural, cellular, and stromal) between nevi and melanoma* ***(Video 3)***
4. *Identify clinical "ABCDE" features of dysplastic nevi and melanoma that make them distinct from benign nevi[1] (ABCDE stands for "asymmetry, border irregularity, color variation, diameter > 6 mm, and evolution")* ***(Videos 3 & 4)***
5. *Describe the four main sub-types of melanoma (superficial, nodular, lentigo maligna, and acral)* ***(Video 4)***
6. *Describe the three main sub-types of benign nevi (junctional, compound, and dermal)* ***(Video 3)***

[1] Note: Despite the fact many dysplastic nevi (also referred to as "atypical melanocytic proliferations", "nevus with architectural disorder", "Clark's nevus", etc.) share clinical and histological ABCDE features with melanoma as discussed in the videos, dysplastic nevi are benign lesions (often called ambiguous melanocytic neoplasms). Although most melanoma arise de novo (i.e. NOT from pre-existing nevi), particularly concerning dysplastic nevi are often removed for their small potential to transform into malignant melanoma.

Authors: Jasmine Rana[1] BA & Arash Mostaghimi[2] MD, MPA, MPH
[1]Harvard Medical School: jasmine_rana@hms.harvard.edu
[2]Brigham and Women's Hospital, Harvard Medical School: amostaghimi@bwh.harvard.edu

Below is a suggested approach to the video module (listed in recommended order):

1. **Review the list of key terms (listed below).** Refer back to these throughout the module. You should able to define/understand the key terms by the end of this module.

   <u>Key Terms</u>

   a. *Distinguish 2 sub-types of keratinocyte carcinoma*
      i. <u>Squamous cell carcinoma (SCC)</u>
         1. *Histological characteristics:*
            a. Pleomorphism
            b. Acanthosis
            c. Hyperkeratosis
            d. Keratin pearls
         2. *Gross features:*
            a. "Scaly, erythematous papule/plaque/nodule"
      ii. <u>Basal cell carcinoma (BCC)</u>
         1. *Histological characteristics:*
            a. Basaloid nests
            b. Peripheral palisading
            c. Retraction artifact
         2. *Gross features (of nodular subtype, most common):*
            a. "Pearly papule/plaque/nodule, rolled borders, telangiectasia"

   b. *Distinguish lesions (grossly and histologically)*
      i. BCC
      ii. SCC
      iii. Benign nevi (moles)
      iv. Atypical (dysplastic) nevi
      v. Melanoma; be aware of four sub-types:
         1. Superficial spreading
         2. Nodular
         3. Lentigo maligna
         4. Acral lentiginous

   c. *Distinguish dysplastic nevi & melanoma from benign nevi*

Authors: Jasmine Rana[1] BA & Arash Mostaghimi[2] MD, MPA, MPH
[1]Harvard Medical School: jasmine_rana@hms.harvard.edu
[2]Brigham and Women's Hospital, Harvard Medical School: amostaghimi@bwh.harvard.edu

i. ABCDE features of dysplastic nevi and melanoma
   1. Asymmetry
   2. Border irregularity
   3. Color variation
   4. Diameter (> 6 mm)
   5. Evolution

ii. Breslow thickness

2. **View concept videos** (can be viewed on any device that supports MP4 files; we suggest viewing them in order; if you cannot view all the videos in one sitting, we suggest you view Videos 1 & 2 together (total viewing time = 26:52 min) followed by Videos 3 & 4 (total viewing time = 27:30 min) as these video pairs roughly cover distinct topics, i.e. keratinocyte skin cancer and melanoma/nevi, respectively)
   a. "Video1_Introduction to skin cancer.mp4" (08:15 minutes)
   b. "Video2_Keratinocyte skin cancer BCC and SCC.mp4" (18:37 minutes)
   c. "Video3_Overview nevi and melanoma.mp4" (13:56 minutes)
   d. "Video4_ABCDE and melanoma.mp4" (13:34 minutes)

3. **Review annotated PowerPoint slides**
   a. "SkinCancer_AnnotatedSlides.pdf"

4. **Complete the review questions (10) to assess your understanding of the preparatory material.** Answers and explanations are provided.
   a. "SkinCancer_ReviewQuestions.pdf"
